# Tom70 Is Essential for PINK1 Import into Mitochondria

**DOI:** 10.1371/journal.pone.0058435

**Published:** 2013-03-05

**Authors:** Hiroki Kato, Qiping Lu, Doron Rapaport, Vera Kozjak-Pavlovic

**Affiliations:** 1 Interfaculty Institute of Biochemistry, University of Tübingen, Tübingen, Germany; 2 Department of Microbiology, Biocentre, University of Würzburg, Würzburg, Germany; Boston University, United States of America

## Abstract

PTEN induced kinase 1 (PINK1) is a serine/threonine kinase in the outer membrane of mitochondria (OMM), and known as a responsible gene of Parkinson's disease (PD). The precursor of PINK1 is synthesized in the cytosol and then imported into the mitochondria via the translocase of the OMM (TOM) complex. However, a large part of PINK1 import mechanism remains unclear. In this study, we examined using cell-free system the mechanism by which PINK1 is targeted to and assembled into mitochondria. Surprisingly, the main component of the import channel, Tom40 was not necessary for PINK1 import. Furthermore, we revealed that the import receptor Tom70 is essential for PINK1 import. In addition, we observed that although PINK1 has predicted mitochondrial targeting signal, it was not processed by the mitochondrial processing peptidase. Thus, our results suggest that PINK1 is imported into mitochondria by a unique pathway that is independent of the TOM core complex but crucially depends on the import receptor Tom70.

## Introduction

Mitochondria are unique organelles which harbor numerous metabolic pathways and supply cells with energy in the form of ATP. The complex biogenesis and dynamics of the mitochondrial network necessitate elaborate quality control measurements to assure that damaged proteins and organelles are eliminated. A dysfunction of mitochondria causes fragmentation of the mitochondrial network and can induce a specific autophagy of mitochondrial fragments (also called mitophagy) [1], [2], [3].

Aberration of mitochondrial quality control has been suggested as a cause of Parkinson's disease (PD) [4], [5], [6]. PD is one of the most common neurodegenerative diseases. Most of the PD cases cannot be attributed to known genetic factors but about 5–10% of the patients suffer from familial PD and bear mutations in specific genes that have been conclusively shown to cause PD [7], [8], [9]. Among these mutated genes are alpha-synuclein (SNCA), leucine-rich repeat kinase 2 (LRRK2), Parkin, DJ1, ATP13A2, and PTEN induced kinase 1 (PINK1) [7], [8], [9]. PINK1, a protein of the outer membrane of mitochondria (OMM), and Parkin, E3 ubiquitin ligase localized in cytosol, are involved in selective clearance of damaged mitochondria. [10]. In normal condition, the precursor of PINK1 (65 kDa) is synthesized in the cytosol and is imported into the OMM. After association with the OMM, PINK1 is further transferred into the inner membrane of mitochondria (IMM) in a membrane potential dependent manner, and is then processed to a 52 kDa mature form by the mitochondrial rhomboid protease in the IMM, PARL [11], [12]. The half life of the mature form of PINK1 is very short (30 min) and it was proposed that the proteasome is involved in its degradation [13]. Hence, under normal conditions the protein level of PINK1 in mitochondria is extremely low. However, when mitochondria are damaged and lose their membrane potential, PINK1 is not imported into the IMM, and rather avoids processing by PARL. PINK1 remains then in the OMM and recruits Parkin, to the OMM where the latter protein induces mitophagy [10].

PINK1 has a predicted mitochondrial targeting signal (MTS) in its amino-terminal region, transmembrane (TM) domain in the middle, and kinase domain in its carboxy -terminal [14]. It has been anticipated that PINK1, like almost all mitochondrial proteins, is synthesized in the cytosol as a preprotein, targeted to the surface of the organelle, and then translocated across the translocase of the OMM (TOM) complex [11], [15], [16]. Subsequently, PINK1 is believed to be imported into the mitochondrial matrix (MTX) by the translocase of the IMM (TIM), and then it was suggested to be cleaved by the mitochondrial processing peptidase (MPP) [17].

In this study, we investigated the import pathway of PINK1 into the mitochondria using a cell-free system. We found that PINK1 is imported into the mitochondria in a membrane-potential dependent manner, is not cleaved by MPP, and that the import receptor Tom70, but not Tom40, is involved in this process.

## Materials and Methods

### Ethics statement

All animal experiments were reviewed and approved by the local authorities (Regierungspräsidium Tübingen, Germany) and were conducted in accordance with the University of Tübingen guidelines and §4 of the German law. We made effort to minimize the number of animals used and their suffering.

### Cell culture

HeLa cells expressing shRNA against TOM proteins under the control of doxycyclin (Dox) were established as described previously [18]. These cells were cultured at 37°C under 5% CO_2_ in RPMI 1640, supplemented with 10% FBS. The knockdown of TOM proteins was induced by adding 1 µg/ml Dox for 7 days or 5 days in the case of Tom40 knockdown cell line.

### Isolation of mitochondria

Mouse liver mitochondria were isolated as described previously [19]. For isolation of mitochondria from tissue culture, HeLa cells in a 10 cm cultured dish were washed with PBS and collected by centrifugation (800× g, 5 min, 4°C). The collected cells were resuspended in buffer A (20 mM Hepes-KOH pH 7.4, containing 220 mM Mannitol, 70 mM sucrose, 1 mM EDTA, 2 mg/ml BSA and 0.5 mM PMSF), and were homogenized by passing 20 times through a 27-gauge needle. This mixture was then centrifuged (800× g, 5 min, 4°C) and the supernatant fraction was centrifuged again (10,000× g, 10 min, 4°C) to obtain the mitochondrial fraction in the pellet. The mitochondria were resuspended in 1 ml buffer B (buffer A without BSA) and then centrifuged again (10,000× g, 10 min, 4°C). This mitochondria pellet was used for import assay.

### Cell-free import assay

Radiolabeled precursor proteins were synthesized by the TNT coupled reticulocyte lysate system (Promega) in the presence of ^35^S-methionine. The radiolabeled proteins were incubated at 15°C (PINK1) or 30°C (pSu9-DHFR and F1β) for various time periods with 25 µg mitochondria in 50 µl of import buffer (10 mM Hepes-KOH pH 7.4, containing 1 mM ATP, 20 mM sodium succinate, 5 mM NADH, 0.5 mM magnesium acetate, 220 mM mannitol, 70 mM sucrose, 0.5 mM PMSF). The mitochondria were then isolated by centrifugation and analyzed by SDS-PAGE and autoradiography. The intensity of bands representing imported proteins was quantified by ImageJ (NIH).

### MPP treatment

Recombinant MPP was purified as described before [20]. ^35^S-methionine-labeled precursor of PINK1 and pSu9-DHFR were incubated for 10 min at 25°C with 2 µg MPP in MPP buffer (50 mM KOAc, 20 mM Tris-HCl, 1 mM DTT, 2 mM MnCl_2_, pH 7.4). EDTA (4 mM final conc.) was added to stop the enzymatic reaction.

### Trypsin treatment of mitochondria

Mitochondria (25 µg) were treated at 0°C for 10 min with 50 µg/ml trypsin in buffer B and then 500 µg/ml of soybean trypsin inhibitor (STI) was added and samples were incubated for 15 min on ice to inactivate trypsin. At the same time, control mitochondria (Tryp−) were incubated with the same amount of trypsin but in the presence of 500 µg/ml STI. After incubation, mitochondria were collected by centrifugation (10,000× g, 10 min, 4°C). Mitochondrial pellet was then used for import assay and membrane potential measurement.

### Membrane potential measurement of isolated mitochondria

3,3′-Dipropylthiadicarbocyanine iodide (diSC3(5)) resuspended in ethanol was added to mitochondrial mixture to a final concentration of 1.5 ng/ml. After 5 min incubation, fluorescence was measured using 651 nm for excitation and 675 nm for emission. The fluorescence intensity of a sample with dye and in the absence of mitochondria was subtracted as a background from the measured fluorescence of the other samples. Three samples were measured for each of the conditions. Measurements were performed in the black plate (Corning) using TECAN Infinite M200 plate reader.

## Results

### Cell-free synthesized PINK1 is imported into the mitochondrial outer membrane

**Figure 1:**
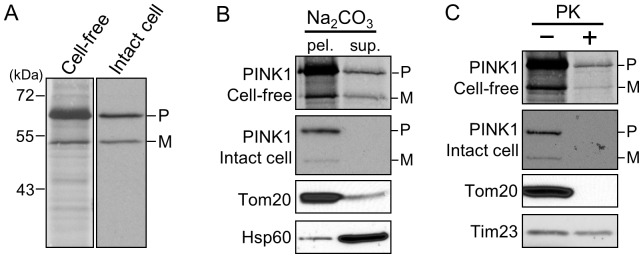
Cell-free import assay of PINK1. (A) Radiolabeled PINK1 was incubated at 15°C for 30 min with mitochondria isolated from HeLa cells. At the end of the import reaction, mitochondria were re-isolated by centrifugation and analyzed by SDS-PAGE and autoradiography (left lane). A plasmid encoding PINK1 was transfected into HeLa cells. Mitochondria were isolated from the transfected cells and analyzed by immunoblotting using PINK1 antibody (right lane). (P) and (M), PINK1 precursor and mature form, respectively. (B) Mitochondria from the cells expressing PINK1 or to which PINK1 was imported were treated with 100 mM Na_2_CO_3_ at 0°C for 30 min, and then the samples were ultracentrifuged to separate the membrane fraction in the pellet (pel.) from soluble proteins in the supernatant (sup.) fraction. Samples were then subjected to SDS-PAGE and autoradiography or immunodecoration with antibodies against PINK1, Tom20 (OMM protein), or Hsp60 (MTX protein). (C) Mitochondria as in part (B) were treated with 100 µg/ml proteinase K at 0°C for 30 min. Samples were then subjected to SDS-PAGE and autoradiography or immunodecoration with antibodies against PINK1, Tom20 (OMM protein) and Tim23 (IMM protein).

To analyse the import pathway of PINK1 into mitochondria, we used a cell-free import assay. Cell-free translated radiolabeled PINK1 precursor was incubated with mitochondria isolated from HeLa cells. Upon import of PINK1 into mitochondria a 52 kDa mature form (M) was detected in addition to the precursor protein (P) (Fig. 1A, left lane). To confirm the identity of the 52 kDa mature form we analyzed PINK1 imported in a cell-free assay and PINK1 expressed in intact cells side by side by SDS-PAGE. Radiolabeled cell-free imported PINK1was detected by autoradiography and expressed PINK1 from intact cells was detected using antibodies recognizing residues 175–250 of PINK1. We observed that both mature forms co-migrated at 52 kDa (Fig. 1A). Furthermore, we analyzed the submitochondrial localization of the imported and expressed PINK1. Alkaline extraction [21] (Fig. 1B) and proteinase K treatment (Fig. 1C) showed that cell-free imported PINK1 is localized in the OMM similarly to PINK1 expressed in intact cells. Taken together, both forms of PINK1 behaved as a membrane-embedded protein exposed to the cytosol.

### PINK1 is not processed by MPP

Most of the MTS-containing mitochondrial precursor proteins are imported into the mitochondria in a process requiring membrane potential and upon their import the MTS is cleaved off by MPP [22]. PINK1 has a predicted MTS in its N-terminal region [14]. Thus, we asked whether PINK1 is imported into the mitochondria through the aforementioned pathway. In these experiments, pSu9-DHFR, the presequence of subunit 9 of the F_1_F_o_ ATPase of *N. crassa* fused to mouse dihydrofolate reductase, was used as a model mitochondrial precursor protein with MTS.

**Figure 2:**
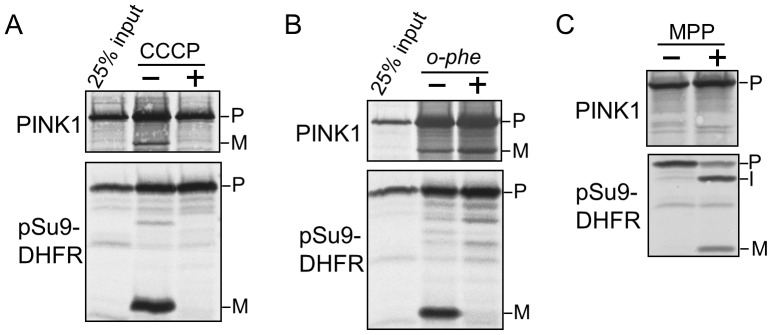
PINK1 is not processed by MPP. (A) Import reactions of radiolabeled PINK1 and pSu9-DHFR were performed in the presence or absence of 20 µM CCCP. Samples were then analyzed by SDS-PAGE and autoradiography. Precursor and mature forms are indicated by P and M, respectively. (B) Mitochondria were pre-incubated with (+) or without (−) 5 mM *o*-phenanthroline (*o*-phe) at 0°C for 10 min, reisolated, and subjected to cell-free import assay of radiolabeled PINK1 and pSu9-DHFR in the presence (+) or absence (−) of 5 mM *o*-phe. Further treatment was as described in part (A). (C) Radiolabeled PINK1 and pSu9-DHFR were incubated with MPP at 25°C for 10 min. The reactions were stopped by adding 4 mM EDTA and samples were subjected to SDS-PAGE and analyzed by autoradiography.

To elucidate the membrane potential dependency we added the uncoupler CCCP to the cell-free import reactions. When CCCP was added, the import of both PINK1 and pSu9-DHFR was interdicted (Fig. 2A). Thus, the membrane potential is necessary for PINK1 processing.

Next, we investigated whether PINK1 is processed by MPP upon its import into mitochondria. MPP is a metallopeptidase which requires divalent metal ions [20]. Thus, to inhibit MPP we added *O*-phenanthroline, a metal ion chelator to the cell-free import reaction. As expected, processing of pSu9-DHFR was inhibited (Fig. 2B, lower panel), but the generation of PINK1 mature form was unaffected (Fig. 2B, upper panel). To support these results, we purified recombinant MPP and incubated it directly with radiolabeled precursors of PINK1 or pSu9-DHFR. Since pSu9-DHFR has two MPP cleavage sites at positions 35 and 66 [23], two fragments representing the intermediate (I) and mature (M) forms appeared upon incubation with MPP (Fig. 2C, lower panel). On the other hand, the proteolytic fragment of PINK1 was not detected after MPP treatment (Fig. 2C, upper panel). Collectively, these results indicate that PINK1 is not cleaved by MPP.

### PINK1 import is independent of Tom40

**Figure 3:**
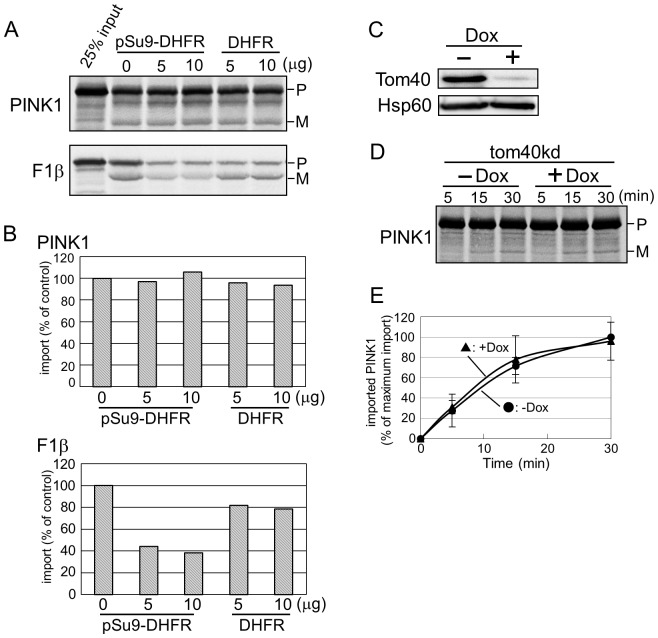
Tom40 is not involved in PINK1 import. (A) Radiolabeled PINK1 and F1β were imported into mitochondria in the presence of the indicated amounts of pSu9-DHFR or DHFR. After import reaction, mitochondria were washed with buffer B. Samples were then analyzed by SDS-PAGE and autoradiography. (B) Bands corresponding to the mature imported protein were quantified. Import into mitochondria in the absence of added protein was taken as 100%. (C) Protein level of mitochondria isolated from either control (−Dox) or Tom40 knockdown cells (+Dox) were analyzed by immunodecoration using the indicated antibodies. (D) Radiolabeled PINK1 was incubated at 15°C with control (−Dox) or Tom40 knockdown (+Dox) mitochondria for the indicated time periods. Samples were then analyzed by SDS-PAGE and autoradiography. (E) Bands corresponding to the mature imported protein were quantified. The amount of mature PINK1 after 30 min import into control mitochondria (−Dox) was set to 100%. The results are the average of three independent experiments ± SD.

The TOM complex is composed of the import channel formed mainly byTom40, the import receptors Tom20, Tom22 and Tom70, and several small Tom proteins [24], [25]. MTS-containing precursor proteins require Tom40 to pass through the OMM. We examined whether Tom40 is essential also for PINK1 import. To that goal, we added high amount of recombinant pSu9-DHFR to the import reaction in order to block the Tom40 channel. As expected, import of F1β, the yeast F1-ATPase beta subunit precursor protein, was interdicted by this treatment (Fig. 3A and B lower panel). In contrast, PINK1 import was not affected by the competition with pSu9-DHFR (Fig. 3A and B upper panel). As a control, we also added DHFR to the import assays and observed that it did not affect the import reactions (Fig. 3A and B). Furthermore, we examined the import into mitochondria isolated from cells where knockdown of Tom40 was performed using doxycycline inducible shRNA cell line [18]. Mitochondria isolated from these Tom40-depleted cells have significant reduced capacity to import VDAC1 [18]. As observed before [18], the levels of Tom40 were efficiently reduced in these cells by adding doxycycline, but the level of Hsp60, a mitochondrial matrix protein, was not affected (Fig. 3C). Therefore, we used Hsp60 to demonstrate that equal amounts of control and Tom40-knockdown mitochondria were used in the import experiment (Fig. 3C). Surprisingly, the reduction of Tom40 did not affect the import of PINK1 (Fig. 3D and E), suggesting that Tom40 is not necessary for PINK1 import.

### Tom70 is important for PINK1 import

**Figure 4:**
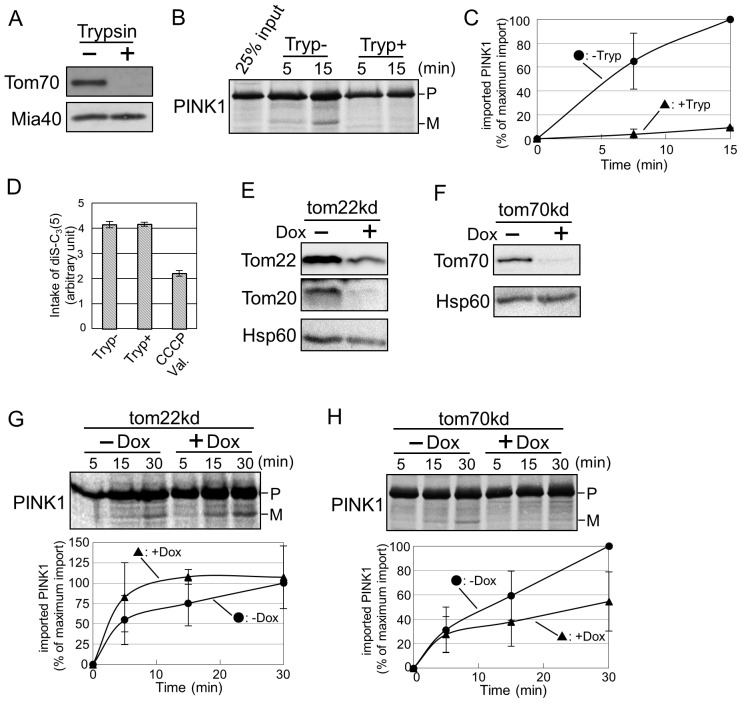
Tom70 is necessary for PINK1 import. (A) Mitochondria isolated from mouse liver were treated at 0°C for 10 min with 50 µg/ml trypsin and then analyzed by western blotting using antibodies against Tom70 (OMM) and Mia40 (IMS) proteins. (B) Radiolabeled PINK1 was incubated at 15°C with intact (Tryp−) or trypsin treated (Tryp+) mitochondria for the indicated time periods. Samples were then analyzed by SDS-PAGE and autoradiography. (C) Bands corresponding to the mature imported protein were quantified. The amount of mature PINK1 at 15 min import into intact mitochondria (Tryp−) was set to 100%. The results are the average of three independent experiments ± SD. (D) The membrane potential of intact mitochondria (Tryp−), trypsin treated mitochondria (Tryp+) and intact mitochondria treated with 1 µM CCCP and 1 µM valinomycin were measured by uptake of fluorescent dye diSC3(5). The results are the average of three experiments ± SD. (E, F) Mitochondria isolated from control cells (−Dox) and fromTom22 (E, +Dox) or 70 knockdown cells (F, +Dox) were analyzed by western blotting using the indicated antibodies. (G, H) Radiolabeled PINK1 was incubated at 15°C for the indicated time intervals with mitochondria isolated from control (−Dox), Tom22-depleted (G, +Dox), or Tom70 depleted cells (H, +Dox). Samples were analyzed by SDS-PAGE and autoradiography (upper panels). Bands corresponding to the mature imported protein were quantified. The amount of mature PINK1 after 30 min import of control mitochondria (−Dox) was set to 100% (Fig. 4G, H lower panels). Graphs represent mean values of three independent experiments ± SD.

The import of most mitochondrial precursor proteins requires initial recognition by import receptors [26], [27], [28]. We examined whether the receptor proteins on the OMM are necessary for PINK1 import. Isolated mitochondria were treated with trypsin in order to remove the exposed domains of the receptor proteins. The receptor protein, Tom70 was indeed removed by such a treatment, but on the other hand, an intermembrane space (IMS) protein, Mia40 remained intact (Fig. 4A). We performed cell-free import assay using trypsin treated mitochondria, and found that the import efficiency of PINK1 was considerably reduced (Fig. 4B and C). Previously, we showed that membrane potential of mitochondria is necessary for PINK1 import (Fig. 2A). Thus, we verified that trypsin treatment did not affect mitochondrial membrane potential using an assay for membrane potential measurement based on the intake of fluorescent dye diSC3(5). We observed that there was no difference in the fluorescence intensity of the trypsin treated (Tryp+) and intact (Tryp−) mitochondria. As expected, the fluorescence intensity was strongly reduced when mitochondria were treated with the uncoupler CCCP and valinomycin, a potassium ionophore, confirming the validity of our measurements (Fig. 4D). Likewise, previous experiments performed with yeast mitochondria showed that trypsin treatment does not affect mitochondrial membrane potential [29].

Tom20, Tom22 and Tom70 were identified as import receptors on the OMM. Tom20 and Tom22 mainly recognize presequence-containing proteins [27], whereas Tom70 is known as a receptor for internal signals in hydrophobic proteins [28]. We aimed to identify the receptors that are involved in PINK1 import using shRNA cell lines of Tom22 and Tom70 (Fig. 4E and F, respectively). Since a major reduction of Tom20 could be seen in shRNA cell line with Tom22 knockdown (Fig. 4E), we used this cell line to analyze the contribution of both receptor proteins. The reduction of Tom20 and Tom22 did not affect PINK1 import (Fig. 4G). In contrast, the import efficiency of PINK1 was reduced by knockdown of Tom70 (Fig. 4H).

## Discussion

In this study we addressed the participation of the TOM complex in PINK1 import using doxycycline inducible shRNA cell lines and by blocking the TOM import channel. We observed that Tom70 is essential for PINK1 import, whereas Tom40 appeared not to be involved in this process. In addition, although we did not obtain complete depletion of Tom22, our results might suggest that Tom20 and Tom22 are also not required for the import of PINK1. Lazarou et al. showed that the TOM core complex, including Tom40, Tom20, and Tom22 forms a complex with PINK1 on depolarized mitochondria [30]. When mitochondria lose membrane potential, PINK1 accumulates on the OMM, and then recruits Parkin. Interestingly, PINK1/TOM complex did not contain Parkin. Hence, it seems that PINK1 associated with the TOM complex lost the ability to recruit Parkin. Accordingly, we speculate that TOM core complex regulates the binding between PINK1 and Parkin rather than import of PINK1 into the mitochondria.

Trypsin pretreatment of mitochondria revealed that the receptors on the OMM are necessary for PINK1 import. Furthermore, using shRNA knockdown cells we found that Tom70 might work as a receptor for PINK1 import. However, the mechanism of the subsequent insertion of PINK1 into the OMM is still unclear. There are two possibilities; PINK1 is directly inserted into the lipid bilayer after recognition by Tom70 or alternatively it is integrated into the membrane with the help of a yet unknown import channel. Several polytopic OMM proteins are imported into the OMM via Tom70 without a clear involvement of Tom40 [31], [32], [33]. It is possible that PINK1 import follows a similar pathway.

PINK1 has a predicted MTS in its N-terminal region and accordingly it was assumed that PINK1 MTS is processed by MPP after import into mitochondria. Greene et al. reported that the knockdown of MPPβ induces increasing amounts of full length PINK1 [17]. However, it is difficult to distinguish whether this is a direct or an indirect effect of MPPβ knockdown. To resolve this problem, we incubated recombinant MPP with PINK1, and observed that MPP did not cleave PINK1. Furthermore, known MPP inhibitor did not affect PINK1 import. Thus, these results indicate that MPP is most likely not directly involved in PINK1 processing.

Taken together, our study indicates that PINK1 is imported into mitochondria by a unique pathway that requires Tom70 but is independent of Tom40 and MPP.
